# Neuroimaging Spectrum of Intracranial Lipomas

**DOI:** 10.7759/cureus.35063

**Published:** 2023-02-16

**Authors:** Tushar Kalekar, Suhas M, Latha P Reddy, Aparna S Prabhu, Purnachandra Lamghare

**Affiliations:** 1 Radiodiagnosis, Dr. D. Y. Patil Medical College, Hospital & Research Centre, Dr. D. Y. Patil Vidyapeeth, Pune, IND

**Keywords:** neuroradiology, neurological symptoms, mri, ct, intracranial lipomas and associations

## Abstract

Background: Intracranial lipomas are rare congenital malformations at characteristic sites. Though largely asymptomatic, some cause significant morbidity. We are studying this for the local population.

Objectives: The purpose of this article is to analyze the spectrum of intracranial lipomas, determine their distribution at characteristic locations, and assess their associations.

Method: A retrospective study of 21 patients diagnosed with intracranial lipomas detected on CT and MRI performed between September 2017 and May 2022 at Dr. D.Y. Patil Medical College, Hospital & Research Center, Pune.

Results: Amongst the 21 patients with intracranial lipomas, the most common location was the pericallosal region seen in 11 patients (n = 11, 52.3%), of which nine were curvilinear type (n = 9), more common than tubular nodular type (n = 2). Other locations (n = 10, 47.7%) were found to be; quadrigeminal cistern in six patients (28.5%), cranial diploic space in two patients (9.5%), one each in interhemispheric falx (4.8%), and cribriform plate (4.8%). Associated anomalies were observed in three patients, which were fronto-ethmoidal encephalocele (n = 1), partial agenesis of the corpus callosum (n = 2), extension with a frontal subcutaneous lipoma (n = 1), and bilateral intraventricular extension (n = 1). Presenting symptoms were headache and dizziness (38.1%), seizures (19%), swelling on the forehead (9.5%), and other non-specific clinical symptoms (33.3%).

Conclusion: Intracranial lipomas are rare congenital lesions that are usually asymptomatic and discovered incidentally. However, lipomas at interhemispheric locations can cause seizure disorders and some present with headaches and dizziness. Few have associations like communication with extracranial swellings and corpus callosum agenesis.

## Introduction

The term intracranial lipomas are used more specifically to describe foci of adipose tissue seen within the intracranial cavity and CSF spaces [1]. 

There are a few studies that have assessed the prevalence and location, but these are done for the western population. Few rare case reports of intracranial lipomas [2] have been reported in India, but a systematic review to assess their prevalence was lacking. Previous autopsy series have shown intracranial lipomas to be very rare lesions. But in a study by J. Gossner, a small retrospective analysis of 50 patients who were undergoing CT for various reasons, nine patients were incidentally found to have small intracranial lipomas. Thus intracranial lipomas may be a more commoner finding lacking clinical relevance [3]. In this study, we want to assess the spectrum of intracranial lipomas, study their clinical presentations, and determine their distribution at characteristic locations in the local population.

Intracranial lipomas are not tumors but are thought to be malformations arising from the differentiation of the aberrant embryological meninx primitiva (the predecessor of the subarachnoid space). This explains why intracranial lipomas are seen in the subarachnoid space and why they have parenchymal abnormalities and traversing structures [4]. Most intracranial lipomas are asymptomatic, but they can present with symptoms like headache and seizures [5,6].

They can occur anywhere, but the common occurrence is at characteristic locations [7] that also determine their specific types - pericallosal lipoma (45%), quadrigeminal lipoma (25%), suprasellar cistern lipoma (15%), cerebellopontine angle lipoma (10%), Sylvian fissure lipoma (5%), and choroid plexus lipoma (rare).

Pericallosal lipomas are divided into tubulonodular and curvilinear types. They are associated with agenesis of the corpus callosum in 50% of cases. Quadrigeminal lipomas are linked to the underdevelopment of the inferior colliculus.

On CT, these appear as non-enhancing masses with fat density. It conforms to adjacent anatomy and has a soft lobulated appearance. Peripheral calcification may be present.

On MRI, they show high signal intensity on T1 and T2, low signal intensity on fat-saturated sequences, and no enhancement on T1 C+ (Gd), and can produce blooming on SWI due to susceptibility artifact.

## Materials and methods

Study design: A retrospective cross-sectional study was conducted on patients who were diagnosed to have intracranial lipomas on computed tomography (CT); or magnetic resonance imaging (MRI) performed during the period from September 2017 to May 2022 at Dr. D.Y. Patil Medical College, Hospital & Research Center, Pune. Twenty-one patients with intracranial lipomas were found when we reviewed the hospital picture archiving and communication system (PACS) and were included in the study.

A waiver letter was obtained from Institutional Ethics Sub-Committee, Dr. D. Y. Patil Medical College, Hospital and Research Centre, Pune, with the ref. No: I.E.S.C./ 247/2022 dated 12/12/2022.

Inclusion and exclusion criteria: Patients of all age groups diagnosed with intracranial lipomas on CT or MRI between September 2017 and May 2022 were included in the study. 

Data collection: Clinical information, including patient age, clinical symptoms, location of intracranial lipoma, and associated intracranial anomaly, were accessed from the hospital picture archiving and communication system (PACS). 

Statistical analysis: Categorical variables were expressed as numbers and percentages, and results were presented in tables.

## Results

Table 1 shows that of the 21 patients studied, eight were male (38.1%), and 13 were female (61.9%). The age at diagnosis ranged from three years to 76 years. 

**Table 1:** Sex distribution

Sex distribution	
Sex	Number
Male	8
Female	13
Total	21

Eleven patients (52.3%) had pericallosal lipomas, curvilinear type (n = 9) being more common than tubular nodular type (n = 2). The rest of the 10 patients had lipomas at other regions, quadrigeminal cistern lipomas being the most common (28.5%), followed by cranial diploic space (9.5%), interhemispheric falx (4.8%), and cribriform plate (4.8%). One case of curvilinear pericallosal lipoma was associated with the fronto-ethmoid encephalocele. Both cases of tubulo-nodular pericallosal lipomas were associated with partial agenesis of the corpus callosum. One case showed an extension with a frontal subcutaneous lipoma, and the other had a bilateral intraventricular extension. The location, frequency of occurrence, and associated anomalies are summarized in Table 2.

**Table 2:** Frequency of location of intracranial lipomas and associated anomalies

Location	Frequency	Percentage	Associated anomalies
Pericallosal lipoma - Curvilinear	9	42.8%	Fronto-ethmoidal encephalocele: 1
Pericallosal lipoma - Tubulo-nodular	2	9.5%	Partial agenesis of the corpus callosum: 2
			Extracranial extension with a frontal subcutaneous lipoma: 1
			Dysplasia of bilateral ACA: 1
			Intraventricular extension: 1
Quadrigeminal Cistern	6	28.5%	Posterior fossa arteriovenous malformation: 1
Interhemispheric falx lipoma	1	4.8%	
Cribriform plate	1	4.8%	
Cranial diploic space lipoma	2	9.5%	

Table 3 shows the frequency of clinical symptoms at presentation in patients with intracranial lipomas. Headache and dizziness (38.1%) and seizures (19%) were the most common presenting symptoms. Among two patients (9.5%) who presented with swelling on the forehead, one patient had a frontal lipoma with extension into an intracranial corpus callosal lipoma; the other had a fronto-ethmoidal encephalocele. The rest of the patients presented with several non-specific clinical symptoms (33.3%). Younger age groups from three to 30 years mostly presented with forehead swellings or seizures, whereas the middle aged and elderly had headache and dizziness as the most common presenting symptoms.

**Table 3:** Frequency of clinical symptoms in intracranial lipomas

Clinical symptoms	Frequency	Percentage
Headache, dizziness	8	38.1%
Seizures	4	19.0%
Swelling on forehead	2	9.5%
Others	7	33.3%

**Figure 1:**
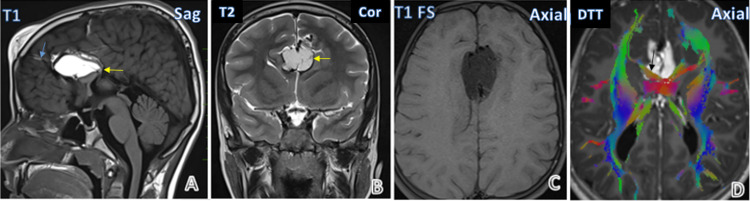
Case gallery Case 1: Corpus callosum tubulonodular lipoma with partial agenesis of corpus callosum (yellow arrow) and connection (blue arrow) with a frontal lipoma (A,B,C). White matter tracts passing through lipoma (black arrow) are seen on tractography (D).

Figure 1 shows a case of tubulonodular pericallosal lipoma with associated anomalies. It showed partial agenesis of the corpus callosum. There is the communication of the intracranial lipoma with frontal subcutaneous lipoma. Lipoma appears bright on both T1WI and T2WI and is suppressed on T1FS images. On Diffusion tensor tractography, white matter tracts can be seen traversing through the lipoma (depicted by the black arrow in Figure 1D). 

**Figure 2:**
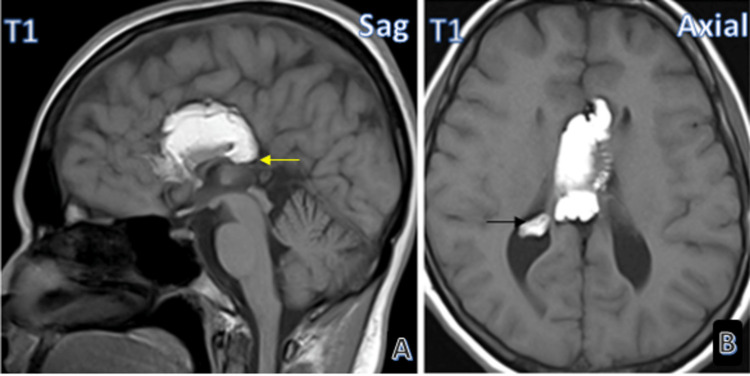
Image gallery Case 2: Tubulonodular lipoma with partial agenesis of corpus callosum (yellow arrow) (A) with intraventricular extension (black arrow) (B).

Figure 2 shows the second case of tubulonodular pericallosal lipoma with associated anomalies. This case, too, had partial agenesis of the corpus callosum and showed intraventricular extension of the pericallosal lipoma. 

**Figure 3:**
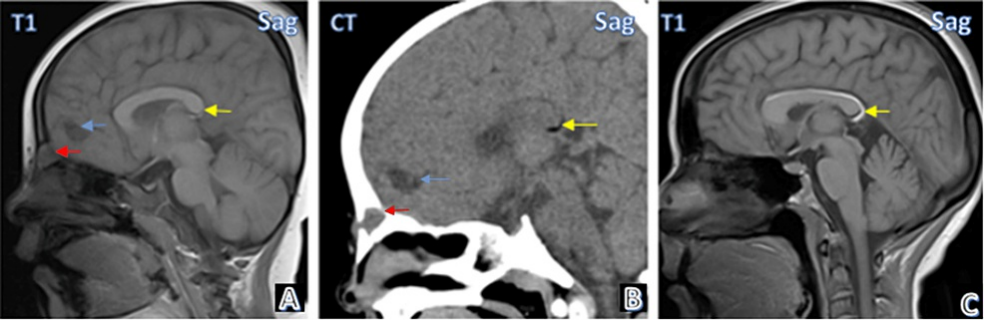
Case gallery Case 3: Frontal meningoencephalocele (red arrow), curvilinear lipoma along posterior aspect of corpus callosum (yellow arrow), focal frontal cystic encephalomalacia (blue arrow) seen on MRI (A) and CT (B). Case 4: Lipoma along the entire extent of the corpus callosum(C). (Yellow arrows depict lipomas in all images)

Figure 3 shows MRI and CT images of the spectrum of intracranial lipomas. Case 3 had curvilinear pericallosal lipoma and was associated with frontal meningoencephalocele and focal frontal cystic encephalocele. 

**Figure 4:**
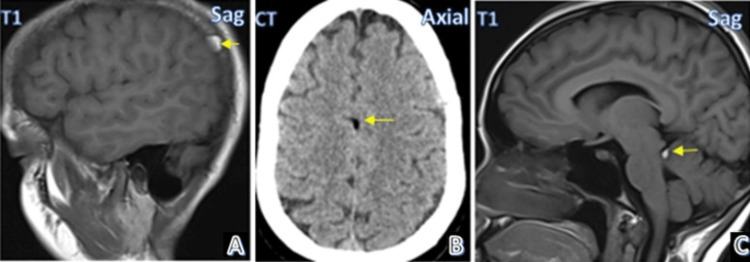
Case gallery Case 5: Lipoma in right parietal space (A). Case 6: Falx lipoma (B). Case 7: Quadrigeminal lipoma (C). (Yellow arrows depict lipomas in all images).

Figure 4 shows MRI and CT images of the spectrum of intracranial lipomas. 

## Discussion

Intracranial lipomas are uncommon congenital lesions [2]. They are a rare finding, found between 0.08% and 0.46% of the time in an autopsy series [3]. Though several theories have tried to explain their cause, the most favorable one is the concept of the subarachnoid space precursor (meninx primitiva), which demonstrates that lipomas arise when a persistent abnormal focus of the meninx primitiva is induced to differentiate into adipose tissue and mature into a lipoma. Lipomas are thus malformations rather than tumors. The meninx primitiva contains primitive perivascular reticuloendothelial, which becomes specialized in storing fat. This also explains the intralesional position of arteries and nerves, the cisternal, subarachnoid location of intracranial lipomas, and the absence of other mesodermal descendants such as muscle [4]. In our study, this explains why, on diffusion tensor tractography, white matter pathways can be seen passing through the lipoma (Figure 1D).

Typically, intracranial lipomas are discovered incidentally. They are usually asymptomatic. However, they can be symptomatic, with epilepsy being the most common finding. By the age of 15, they develop epilepsy, which is nearly always partial. The pathophysiology of seizures appears to be interhemispheric disconnection [5]. Dementia, an elevated intracranial pressure, and hemiparesis are less frequent symptoms. In a clinical study of 14 patients with intracranial lipomas, the reasons for admission were headache (50%), trauma (21.5%), epilepsy (21.5%), and mass effect symptoms (7%) [6]. Presenting symptoms in our 21 patients were headache and dizziness (38.1%), seizures (19%), swelling on the forehead (9.5%), and non-specific clinical symptoms (33.3%). Younger patients mostly presented with forehead swellings or seizures, whereas headache and dizziness were the most common presenting symptoms in the middle aged and elderly.

Intracranial lipomas are present at characteristic locations. They are comprised of the interhemispheric (45%), quadrigeminal/superior cerebellar (25%), suprasellar/interpeduncular (14%), cerebellopontine angle (9%), and sylvian fissure (5%). In a study of 24 patients with intracranial lipomas, 18 were tubulonodular, and six were curvilinear [7]. In our study, the intracranial locations of the lipomas were pericallosal (n = 11, 52.3%), curvilinear (n = 9) being more common than tubular nodular (n = 2), and other regions (n = 10, 47.7%): quadrigeminal cistern (28.5%), cranial diploic space (9.5%), interhemispheric falx (4.8%), and cribriform plate (4.8%).

Interhemispheric lipomas can rarely be associated with the subcutaneous extension. Most of such cases in the literature are in the pediatric population [8-14]. In our study, associated anomalies were observed in three patients. Associated anomalies: fronto-ethmoid encephalocele (n = 1), partial agenesis of the corpus callosum (n = 2), extension with frontal sub cutaneous lipoma (n = 1), and bilateral intraventricular extension (n = 1).

A biopsy is not required as CT and MR findings are characteristic of intracranial lipomas.

Limitation: As this is a retrospective study and only patients who underwent CT or MRI were considered, it may not reflect the true incidence of intracranial lipomas and their health burden. As intracranial lipomas are usually asymptomatic and many patients have not undergone CT or MRI, many cases go undetected. As the study was done in a single institution, it represents the local population of the Pune area and may not reflect the wider national population. 

## Conclusions

Intracranial lipomas are rare congenital lesions that are usually asymptomatic and incidental findings. However, lipomas at interhemispheric locations can cause seizure disorders, and some can present with headaches and dizziness. It is important to look for other associated anomalies as their detection significantly impacts patient management, as demonstrated in the case of intracranial extension of a frontal lipoma with tubulonodular corpus callosal lipoma. Other associations are also seen, like an intraventricular extension of lipomas and corpus callosum agenesis.
